# Urinary volatilomics using liquid-liquid extraction and gas chromatography-mass spectrometry (GC-MS) combined with machine learning algorithms as a tool for diagnosis and surveillance of urothelial bladder cancer

**DOI:** 10.1038/s44276-026-00244-8

**Published:** 2026-08-07

**Authors:** Ignacio Deza, Ben de Lacy Costello, Natalia Drabińska-Fois, Paul White, Norman Ratcliffe, Henry Lazarowicz, Chris Probert

**Affiliations:** 1 Department of Computer Science and Creative Technologies, University of the West of England, Coldharbour Lane, Bristol, UK; 2 Centre for Biomedical Research, University of the West of England, Coldharbour Lane, Bristol, UK; 3 Food Volatilomics and Sensomics Group, Faculty of Food Science and Nutrition, Poznan University of Life Sciences, Poznań, Poland; 4 Department of Engineering, Design and Mathematics, University of the West of England, Coldharbour Lane, Bristol, UK; 5 Department of Urology, Royal Liverpool University Hospital, Liverpool University Hospitals, NHS Trust, Liverpool, UK; 6 Department of Molecular and Clinical Cancer Medicine, Institute of Systems, Molecular and Integrative Biology, University of Liverpool, Liverpool, UK

## Abstract

**Background:**

Bladder cancer is the 11th most common cancer in the United Kingdom, with approximately 10,500 new cases annually. Diagnosis and surveillance typically involve cystoscopy, an expensive, time-consuming, and uncomfortable procedure which has encouraged efforts to identify biomarkers, particularly in urine, given its direct contact with malignant tissue.

**Methods:**

Urine collected from 100 participants (50 bladder cancer patients, 50 controls) was subjected to solvent extraction followed by gas chromatography-mass spectrometry (GC-MS) to determine potential volatile and semi-volatile biomarkers. The results were analysed using classical univariate statistics and machine learning methods. Five machine learning algorithms were evaluated, with recursive feature elimination (RFE) identifying optimal biomarker panels.

**Results:**

Machine learning with XGBoost achieved area under the receiver operating characteristic curve (AUROC) of 0.869 (95% CI: 0.740–0.988), representing a significant improvement over the classical statistical approach (AUROC 0.752). An 8-metabolite panel achieved balanced sensitivity and specificity of 85%, or 95% sensitivity with 70% specificity when optimised for screening.

**Conclusions:**

The findings indicate that solvent extraction of urine shows promise for isolating putative biomarkers of bladder cancer. Employing machine learning achieved diagnostic accuracy potentially suitable for clinical deployment as a non-invasive bladder cancer detection tool.

## Introduction

Urothelial bladder cancer (UBC) is one of the most common cancers in Western societies [1], accounting for *circa* 213,000 deaths worldwide annually [2] and is one of the most expensive malignancies to manage per patient from diagnosis to death [3]. Hence numerous studies have aimed to define specific UBC biomarkers to facilitate diagnosis. Applications of metabolomics for cancer diagnosis have been extensively reviewed [4–7], using for instance, liquid chromatography-mass spectrometry (LC-MS) [8], nuclear magnetic resonance spectroscopy (NMR) [9], and gas chromatography-mass spectrometry (GC-MS) [10].

Over recent years, metabolic profiling using volatile organic compounds (VOCs), termed volatilomics, has gained increasing attention for disease diagnoses [11–14]. Many VOCs are products of cell metabolism and can be found in bodily fluids including urine at various concentrations [15]. Given that cancer development is associated with specific metabolic changes and consequently altered VOC profiles, volatilomics has been identified as a promising tool for early detection of pathological states [11, 12, 16].

To date, 444 VOCs have been detected in urine samples from healthy humans, highlighting the range of compounds that can potentially be linked to disease states [15]. Breath contains more identified VOCs (1488) which highlights the greater number of studies that have focused on breath vs. urine biomarkers linked to disease states. However, urine offers advantages over breath including reduced environmental contamination risk and fewer challenges with sampling and storage [17]. Promising applications of urine VOC analyses have been reported, particularly for prostate, bladder, urinary tract-related, and gastrointestinal cancers [16, 18–21]. Urine is favoured for metabolomic analyses due to its availability in large volumes and high levels of patient acceptability due to the non-invasive collection method which minimises patient discomfort, even with repeated sampling [22].

VOC analysis employs various analytical approaches, with GC-MS representing the gold standard [23]. A crucial step in GC-MS analysis is VOC extraction and preconcentration. Most methods rely on sorption onto sorbents, with solid phase microextraction (SPME) being most commonly used [24]. Other absorption-based techniques used include thermal desorption, stir bar sorptive extraction, solid-phase dynamic extraction, single-drop microextraction and vacuum-assisted sorbent extraction [25]. However, SPME has several drawbacks including the limited compound range that can be absorbed due to restricted coating availability. Although new coatings and geometries with higher absorption surfaces have been developed, problems remain [24], particularly for polar VOCs. Moreover, displacement and saturation effects, high fibre cost and fragility, and instability of sorbed VOCs during transport limit SPME applicability in routine diagnostic testing. Hence exhaustive extraction methods such as liquid-liquid extraction (LLE) merit investigation. Recent reports demonstrate that LLE can successfully detect large numbers of VOCs from urine [26, 27].

To date, protein-based urinary biomarkers such as nuclear matrix protein 22 (NMP22), bladder tumour antigens (BTA), and carbonic anhydrase IX (CAIX) have been proposed [28]; however, their sensitivity and specificity remain insufficient to replace cystoscopy [29] and further biomarker discovery studies are needed. Several studies have explored VOCs in urine from UBC patients. An early 1999 study showed formaldehyde (analysed using selected ion flow tube mass spectrometry) was a potential UBC biomarker [30]. Other potential biomarkers were proposed by Jobu et al. [31] using a needle trap device (NTD) for VOC pre-concentration, though their study included only 9 UBC patients. Cauchi et al. [32] reported elevated concentrations of hexanal, benzaldehyde, butyrophenone, 3-hydroxyanthranilic acid, benzoic acid, trans-3-hexenoic acid, cis-3-hexenoic acid, and 2-butanone in UBC patients compared to healthy subjects, achieving 89% overall accuracy (90% sensitivity, 88% specificity) using SPME. More recently, Lett et al. [16] used SPME to identify eight and six VOCs as diagnostic and surveillance biomarkers with 77% and 80% accuracy, respectively. Tyagi et al. [33] utilised thermal desorption coupled with GC-TOF-MS to analyse 15 bladder cancer samples vs. 36 healthy controls and obtained an AUC of 0.81. This study found 13 VOCs that were associated with bladder cancer vs. controls. In a recent study Carapito et al. [34] measured urine from 87 Bladder cancer patients and 90 controls using HS-SPME coupled with GC-MS and obtained an AUROC of 0.872 with an 8 VOC panel.

Traditional biomarker discovery employs univariate statistical methods that identify individual metabolites showing large between-group differences [16, 21]. This implicitly assumes that disease signatures manifest as dramatically altered individual compounds. This approach underpins most prior VOC studies, which report specific biomarkers with differing concentrations in cancer patients. Machine learning methods can model complex non-linear interactions between multiple metabolites, potentially revealing coordinated metabolic patterns that are invisible to univariate analysis.

Many prior studies employed SPME and univariate or simple multivariate statistical methods to identify individual biomarkers. However, analytical challenges associated with SPME may limit compound detection, whilst univariate statistical approaches cannot detect diagnostic signals residing in coordinated patterns across multiple metabolites. None of these prior studies used LLE for VOC extraction or comprehensively compared classical statistical approaches with machine learning methods capable of modelling metabolite interactions.

Therefore, this study had dual aims: 1. to assess LLE as an alternative extraction method addressing SPME limitations, and 2. to compare classical univariate statistical analysis with machine learning approaches for identifying urinary VOC signatures of UBC.

## Materials and methods

### Participants and sample collection

Urine samples from 50 healthy individuals and 50 patients with UBC were used for this study. The 50 healthy patients comprise two sub-groups of: H – 31 individuals who have never had UBC and R – 19 patients in complete remission (no evidence of disease); the 50 patients with UBC comprise two sub-groups of: R-UBC – 36 patients with a recurrence of UBC and N-UBC – 14 patients with a new diagnosis of UBC. Patients were recruited from urology clinics at the Royal Liverpool and Broadgreen University Hospitals NHS Trust between August 1, 2016 and July 31, 2018. These patients represented two clinical opportunities: (i) diagnostic patients undergoing cystoscopy to investigate visible and non-visible haematuria; (ii) surveillance patients with previously diagnosed and treated UBC attending for follow-up cystoscopy as recommended by the current national guideline. Urine samples were collected prior to cystoscopy in the urology clinic. Samples were stored in a refrigerator until the end of the clinic (max 6 h) and then transported to the Liverpool Tissue Bank for long-term storage at −80 °C. Samples were obtained from Liverpool Bio-Innovation Hub (LBIH) Biobank. The LBIH Biobank has Research Tissue Bank status and is licensed by the Human Tissue Authority (HTA). The collection and storage of bio samples have been ethically approved by the North-West 5 Research Ethics Committee. Samples were stored in 2 mL aliquots in 5 ml polypropylene Eppendorf tubes at −80 °C after collection and defrosted in the fridge at 4 °C before analysis. Samples were transported to the analytical laboratory at UWE packed in dry ice and then stored at −80 °C until solvent extraction. The samples were solvent extracted in a single batch and extracts were stored at −20 °C prior to analysis via GC-MS. Samples were run within 1 week of solvent extraction. The characteristics of participants are summarised in Table 1. Table S1 gives the comorbidities for the patients showing there were a range of co-morbidities listed for the cancer (9 patients) and control (24 patients) groups. Samples were not excluded on the basis of co-morbidities as these reflect the likely clinical cases. Clinically relevant controls, patients attending the same haematuria clinics or cancer surveillance clinics having previously had bladder cancer were selected for this study.

**Table 1:** The clinical characteristics of participants in this study.

	Cancer		Control	
	New UBC diagnosis	Recurrence of UBC	Non-UBC haematuria	No recurrence of UBC
Number of participants				
General	15	35	30	20
Male	11	26	20	17
Female	4	9	10	3
Age median (Range) years				
General	65 (53–80)	73 (38–90)	73 (35–90)	69 (51–90)
Male	66 (53–80)	74 (38–90)	73.5 (35–90)	70 (51–90)
Female	62.5 (58–65)	71 (59–84)	64.5 (41–80)	66 (59–68)
Tobacco smoking, frequency (%)				
Current Smoker	3 (20%)	1 (3%)	1 (4%)	2 (10%)
Ex-Smoker	7 (47%)	20 (57%)	17 (56%)	12 (60%)
Non-Smoker	2 (13%)	1 (3%)	12 (40%)	5 (25%)
Information not given	3 (20%)	13 (37%)		1 (5%)
Indication for investigation (%)				
Haematuria clinic diagnostic cystoscopy (visible)	7 (47%)		17 (57%)	
Haematuria clinic diagnostic cystoscopy (nonvisible)	1 (7%)		5 (16.5%)	
Haematuria clinic diagnostic cystoscopy (other or unknown)	2 (13%)		3 (10%)	
Non-haematuria clinic diagnostic cystoscopy			5 (16.5%)	
Cancer surveillance programme	5 (33%)	35 (100%)		20 (100%)
Procedure performed, frequency (%)				
Flexible cystoscopy (+/− biopsy)	2 (13%)	7 (20%)	27 (90%)	17 (85%)
Cystoscopy (+/− general anaesthesia + /− biopsy)		2 (6%)	2 (7%)	
TURBT	13 (87%)	26 (74%)	1 (3%)	3 (15%)
Tumour stage as per TNM, frequency (%)				
Ta	10 (66%)	20 (57%)	N/A	N/A
T1	1 (7%)	4 (11%)	N/A	N/A
T2 (non-specified)		1 (3%)	N/A	N/A
T2a	4 (27%)	3 (9%)	N/A	N/A
Unknown		7 (20%)	N/A	N/A
Tumour grade, frequency (%)				
G1			N/A	N/A
G2 not specified		3 (9%)	N/A	N/A
G2 low grade	4 (27%)	9 (25%)	N/A	N/A
G2 high grade	1 (7%)	3 (9%)	N/A	N/A
G3	10 (66%)	15 (43%)	N/A	N/A
G3 carcinoma in-situ		4 (11%)	N/A	N/A
Unknown		1 (3%)	N/A	N/A

### Volatile organic compounds analysis

VOCs from urine were analysed following a previously optimised and validated method [26]. Briefly, 2 mL of 1 M sulfuric acid were added to 2 mL of urine in the presence of 0.2 g of sodium sulphate, followed by vortex mixing for 1 min using a Clifton Cyclone Vortex Mixer (Fisher Scientific, Loughborough, UK). Next, 4 mL of dichloromethane (DCM) were added and vortex mixed again. The layers were separated by centrifugation for 1 minute at 3500 rpm using a Beckman Coulter Aliegra X-22R Centrifuge (Brea, CA, USA). The organic layer was dried with anhydrous sodium sulphate and evaporated to dryness in a dry heating block set at 40 °C. The residue was stored at −20 °C until analysed. Prior to the analysis, the residue was dissolved in 10 µL of dichloromethane and 2 µL were immediately manually injected into the inlet of the GC.

The samples were analysed using a Hewlett Packard HP5890 series II GC coupled to an HP5971 single quadrupole Mass Selective Detector (MSD) (Hewlett Packard, Bracknell, UK) and an Agilent Technologies 7820 A GC with Agilent 5977B MSD (Agilent Technologies, Santa Clara, USA. The separation was performed on a Stablewax DA capillary column (30 m × 0.25 mm × 0.25 μm, Restek, Benner Circle, Bellefonte, PA, USA). This column was selected as it is an application specific column for measuring volatile free-acids and many of the compounds identified in the urine had free carboxylic acid groups [26]. The carrier gas was 99.9995% pure helium (AirProducts, Crewe, UK) with a constant flow rate of 1.2 mL/min. The GC oven programme was as follows: 40 °C held for 2 min, ramping at 4 °C/min to the final temperature of 245 °C and then held for 6 min. The injection port temperature was 245 °C.

Mass spectra were acquired in full scan mode with a scan range of m/z 35–450. The MS transfer line and ion source temperatures were 280 °C and 180 °C, respectively.

### Data processing and preliminary analysis

Data obtained from GC-MS chromatograms were processed using the XCMS Online platform (Version 3.7.1) [35]. The chromatograms underwent retention time alignment and feature detection which yielded 916 VOC features. This preliminary analysis examined whether disease history influenced VOC profiles by comparing healthy controls (H, *n* = 31) with patients in complete remission (R, *n* = 19), and new (N-UBC *n* = 14) with recurrent (R-UBC, *n* = 36) bladder cancer patients. No substantial differences were observed between these subgroups; consequently, analyses proceeded as a binary comparison between cancer (N-UBC and R-UBC combined, *n* = 50) and non-cancer (H and R combined, *n* = 50) groups. Python (Python 3.12.11) and a variety of standard Python Machine Learning libraries (pandas: 2.3.1, numpy: 2.3.3, matplotlib: 3.10.0, sklearn: 1.7.1, xgboost: 3.0.1, scikit-learn: 1.7.1) were used for this study [36–38]. Two different statistical methods were employed to identify urinary VOC biomarkers for bladder cancer. Classical univariate statistics and Tree based machine learning methods. Classical statistics approaches assessed each metabolite feature independently, testing for between-group differences under the assumption that features do not interact or, at most, interact linearly through additive effects.

Tree-based ML ensemble methods were selected for their capacity to partition metabolic space through recursive, non-linear decision boundaries whilst maintaining interpretability through feature importance metrics. These algorithms naturally handle varying scales, are robust to outliers, and can model arbitrary interactions without requiring pre-specification of interaction terms. The classical univariate statistical models provided baseline comparisons, representing the independence and linearity assumptions inherent in classical statistical approaches.

Both classical statistical and machine learning approaches were subjected to identical validation frameworks to ensure fair comparison and rigorous performance estimation. The 5-fold cross-validation strategy was employed and repeated 10 times meaning each model is trained and tested 50 times on different data partitions, with performance metrics averaged across all iterations [39]. All training-validation splits employed stratified sampling to maintain equal class representation in the training and validation sets. This approach reduces the risk of overfitting and ensures that reported performance reflects the generalisation capacity rather than random data splits—a critical consideration given the modest sample size (*n* = 100). Whilst the limited sample size constrains the precision of the performance estimates and the ability to detect rare metabolic patterns, the cross-validation framework provides robust estimates of model behaviour within this cohort.

### Classical statistical approach

The XCMS data comprises of the m/z values of fragment ions from the total ion chromatograms that significantly differ between the cancer and control group alongside the retention time. There is also the abundance of each fragment ion in each individual sample along with the mean and standard deviation of the abundance across the cancer and control samples. There were 916 individual features identified which differed between the cancer and control chromatograms. The m/z features have an associated p-value which indicates the statistical significance of differences in metabolite abundance between the cancer and control groups. The m/z values also have an associated fold change (and log_2_ fold change) which is a measure of the difference in intensity of the features between the control and cancer groups. Each m/z feature also has an associated tstat value which represents Welch’s two-sample t-statistic which measures the significance of the difference in mean intensities for a specific metabolite between the cancer and control groups. The value of tstat can be positive (indictaing that the analyte has a higher intensity in the cancer group) or negative (indicating that the analyte has a higher intensity in the control).

**Fig. 1:**
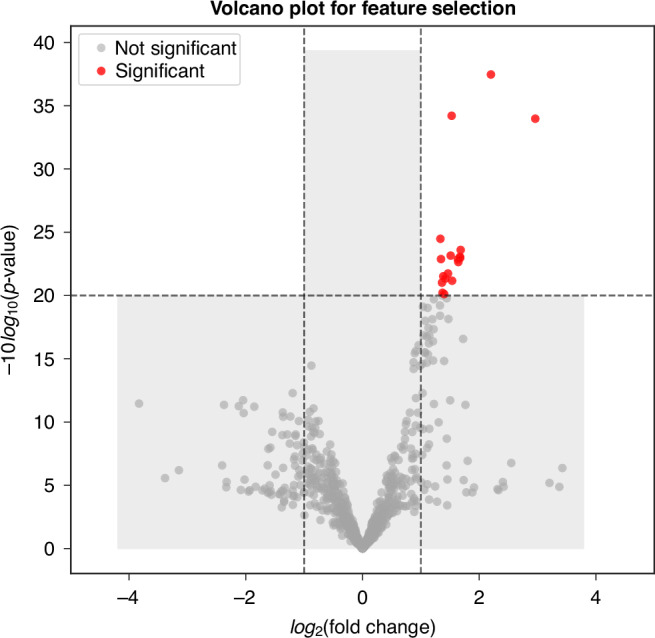
Volcano plot identifying differentially abundant metabolites between the cancer and control groups. Features meeting both criteria, −10×log₁₀(p) > 20 and log₂(fold change) > 1 (*n* = 18 features, highlighted as red circles) were selected for classical statistical modelling.

Volcano plots (Fig. 1) were constructed plotting -10×log₁₀(p-value) against the square root of log₂ (fold change) to simultaneously visualise statistical significance and effect size. Candidate biomarkers were required to satisfy dual criteria: statistical significance [−10×log₁₀(p) > 20, corresponding to *p* < 10⁻²⁰] and substantial effect size [log₂(fold change) > 1, representing ≥2-fold abundance change]. This threshold ensures identified metabolites demonstrate both reproducible differences and sufficient dynamic range for clinical implementation. A square root transformation was undertaken to attenuate the influence of extreme values, a consideration when metabolite concentrations span multiple orders of magnitude. However, once statistically significant features were identified via the transformed data, all subsequent logistic regression modelling used raw, untransformed feature values. This approach was adopted to maintain methodological consistency with the machine learning pipeline (which used untransformed data throughout) to facilitate direct comparison. A logistic regression model constructed from the XCMS data confirmed negligible performance differences between transformed and untransformed features, indicating that the transformation primarily aids visual interpretation of the volcano plot rather than substantively altering the diagnostic capacity.

Between-group differences were assessed using Welch’s [40] t-test, which accommodates the unequal variances expected across heterogeneous disease states without assuming equal population variances. Multiple testing correction was evaluated using several methods including Benjamini-Hochberg [41]; however, as these corrections did not alter the set of significant features, uncorrected p-values are presented.

The diagnostic utility of significant features was evaluated via logistic regression, modelling cancer probability as a linear function of VOC abundance. Model performance was assessed using 5-fold cross-validation repeated 10 times.

### Machine learning approach

For the machine learning analyses, no data transformation was undertaken. The 916 features presented the dual challenge of computational cost and overfitting risk where the number of predictors exceeds the sample size. A systematic two-stage data reduction strategy was implemented to address both concerns whilst preserving diagnostic information.

First, redundant features were identified and consolidated. Pearson correlation coefficients were calculated for all feature pairs, and when correlation exceeded 0.98, indicating near-perfect collinearity, one feature from the pair was removed. This threshold was determined empirically to balance redundancy removal with information retention; thresholds below 0.95 eliminated excessive features whilst those above 0.99 retained near-duplicate compounds. The 0.98 threshold eliminated redundant chemical information (likely representing the same compound detected at marginally different retention times or closely related isomeric species) whilst ensuring computational tractability. From each correlated pair, the feature with higher variance was retained, as higher variance implies greater dynamic range and potentially greater discriminatory power; when variances were equal, selection was random. This preprocessing reduced the feature set by approximately 15%, from 916 to 765 features, improving computational efficiency whilst retaining all functionally unique metabolic signals.

Second, Recursive Feature Elimination (RFE) with XGBoost was employed to identify the minimal metabolite panel achieving optimal discrimination [42]. RFE is a backward selection algorithm that iteratively removes the least informative features and retrains the model, effectively optimising for metabolites that collectively, rather than individually, distinguish disease states.

To ensure reproducibility and identify stable features resistant to sampling variability, the RFE process was repeated 30 times with different random initialisations. Features appearing in ≥80% of the 30 RFE iterations were designated as stable candidates, yielding a subset of 33 metabolites. Hyperparameter optimisation via grid search was then performed on this 33-feature set to identify optimal XGBoost parameters. Hyperparameter optimisation was performed via nested cross-validation [43]: stratified k-fold cross-validation within the outer training folds, ensuring no data leakage between hyperparameter selection and performance estimation. Using these optimised parameters, nested cross-validated recursive feature elimination (RFECV) systematically reduced the feature set from 33 down to 2, evaluating model performance at each step via area under the receiver operating characteristic curve (AUROC). The 8-feature subset demonstrating peak AUROC was selected as the optimal metabolite panel, representing the point at which AUROC peaked before declining due to insufficient information or increasing due to noise from excess features. A final hyperparameter optimisation was conducted on this 8-feature set to determine the optimal parameters for final model training.

Five algorithms were trained on the optimised 8-feature panel: three tree-based ensemble methods (AdaBoost [44], Random Forests [45], XGBoost [46]) and two linear baseline methods (Logistic Regression, Naïve Bayes). All algorithms underwent hyperparameter optimisation via grid search cross-validation. To assess whether the full 8-metabolite panel was necessary or whether subsets sufficed, features were entered sequentially in order of permutation importance [47] (derived from the optimised XGBoost model), with diagnostic accuracy evaluated at each step through cumulative feature addition.

All model performance estimates were derived using 5-fold cross-validation repeated 10 times, a validation strategy that randomly partitions the dataset into training (80%) and testing (20%) subsets and repeats this process 50 times with different random splits.

Three clinically relevant operating points were defined for each model [48]: (1) Youden’s index, maximising the sum of sensitivity and specificity to achieve balanced diagnostic accuracy; (2) a screening-optimised threshold, prioritising sensitivity to minimise false negatives—appropriate when the primary concern is ensuring no cancer cases are missed, even at the cost of additional confirmatory testing; and (3) a diagnosis-optimised threshold, prioritising specificity to minimise false positives—appropriate for confirmatory testing where unnecessary invasive procedures must be avoided.

## Results

### Classical statistical analysis

**Fig. 2:**
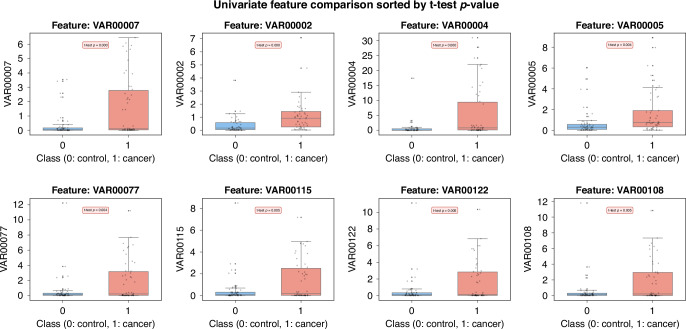
Box-and-whisker plots for the eight most significant VOC features for differentiating bladder cancer and control groups using classical statistic models. The control group in each plot is highlighted in blue and the cancer group in red, the *p*-value for each individual feature is highlighted in orange at the top of each plot.

Volcano plot analysis identified 18 VOC features satisfying both statistical significance (*p* < 10⁻²⁰) and substantial effect size (≥2-fold abundance changes between cancer and non-cancer groups) criteria (Fig. 1). The 18 features had m/z values and retention times as follows: VAR00002 m/z 70.05, RT 33.68, VAR00004 m/z 99.1, RT 28.60, VAR00005 m/z 113.1, RT 28.60, VAR00007 m/z 43.1, RT 28.59, VAR00008 m/z 126.15, RT 28.60, VAR 00027 m/z 43.1, RT 33.68, VAR00060 m/z 57.05, RT 30.6, VAR00065 m/z 116.1, RT 40.75, VAR00070 m/z 92.05, RT 40.75, VAR00073 m/z 100.1 RT 57.65, VAR00077 m/z 50.1, RT 35.54, VAR00087 m/z 53.05, RT 43.37, VAR00090 m/z 39.1, RT 35.53, VAR 00092 m/z 257.25 RT 52.29, VAR00106 m/z 106.1, RT 35.22, VAR00108 m/z 82.15, RT 36.6, VAR00115 m/z 70.05, RT 30.94, VAR00122 m/z 49.08, RT 36.6. All features were upregulated in the cancer group except for VAR00065 and VAR00090. When all 18 features were incorporated into a logistic regression model and evaluated via 5-fold cross-validation repeated 10 times, diagnostic performance reached an AUROC of 0.752 ± 0.096 (95% CI: 0.560–0.908). Box plot visualisation of the eight most statistically significant features (Fig. 2) demonstrated varying degrees of separation between cancer and non-cancer samples.

Performance across three clinically relevant operating points is presented in Table 2. The overall performance of the model is of about 75% with the Youden’s point having an acceptable sensitivity of about 70% and a very high Specificity of about 84%.

**Table 2:** Classical statistical approach performance across three operating points (Youden’s Index, Screening-optimised and Diagnosis optimised) compared to best performing machine learning approach XGBoost (values in bold).

Operating point	Use case	Threshold	Sensitivity (%)	Specificity (%)	AUROC
Youden’s Index	Balanced diagnosis	0.533 ± 0.166, **0.519** ± **0.235**	70.2 ± 19.4, **85.2** ± **11.2**	84.4 ± 17.1, **84.6** ± **10.4**	0.752 ± 0.096, **0.869** ± **0.068**
Screening-optimised	Minimise false negatives	0.392 ± 0.085, **0.314** ± **0.217**	83.4 ± 14.8, **95.2** ± **5.7**	62.8 ± 13.6, **69.6** ± **15.1**	0.752 ± 0.096, **0.869** ± **0.068**
Diagnosis-optimised	Minimise false positives	0.566 ± 0.142, **0.752** ± **0.217**	59.6 ± 11.5, **66.2** ± **16.0**	88.8 ± 13.4, **93.2** ± **7.6**	0.752 ± 0.096, **0.869** ± **0.068**

Values are mean ± SD from 50 cross-validation evaluations.

### Machine learning approach

Recursive feature elimination with XGBoost, following correlation-based preprocessing and stability selection across 30 independent runs, identified an optimal 8-metabolite panel. This panel represents the minimal feature set achieving maximum discrimination, selected explicitly for collective diagnostic performance rather than individual effect sizes. Notably, these eight features differ from those identified by volcano plot analysis, reflecting the fundamental difference between univariate significance testing and multivariate pattern recognition.

**Fig. 3:**
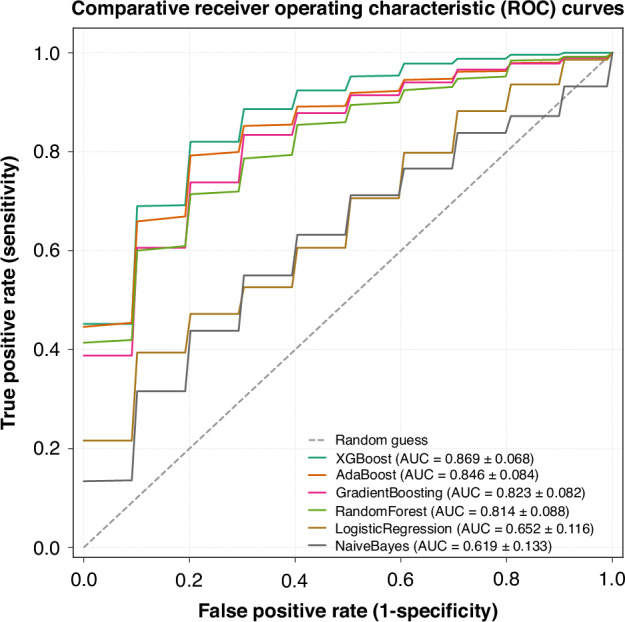
The Receiver operating characteristic (ROC) curves for all machine learning algorithms used to differentiate control groups from bladder cancer patients. The figure shows there is clear separation between tree-based (XGBoost, AdaBoost, Gradientboosting and Random Forest) and linear methods (Logistic Regression and NaiveBayes) showing that the modelling of non-linear metabolite interactions provides better classification of the cancer and control groups.

When evaluated across five machine learning algorithms using 5-fold cross-validation repeated 10 times, tree-based ensemble methods substantially outperformed linear baseline models (Fig. 3). XGBoost achieved the highest performance with AUROC of 0.869 ± 0.068 (95% CI: 0.740–0.988), followed by AdaBoost at 0.846 ± 0.085, Gradient Boosting at 0.823 ± 0.082, and Random Forest at 0.814 ± 0.088. In contrast, linear methods showed inferior performance: Logistic Regression achieved only 0.652 ± 0.116 AUROC, whilst Naïve Bayes reached 0.619 ± 0.133.

**Fig. 4:**
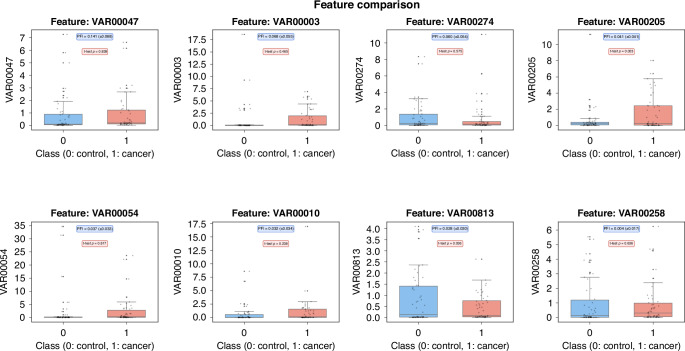
Box-and-whisker plots for the eight most significant VOC features for differentiating cancer and control groups using the best performing machine learning model XGBoost. The control group in each plot is highlighted in blue and the cancer group in red, the p-value for each individual feature is highlighted in orange at the top of each plot and the permutation feature importance is highlighted in blue.

The performance gap between the best tree-based method (XGBoost: 0.869) and the best linear method (Logistic Regression: 0.652) represents a 21.7 percentage point improvement in AUROC, directly quantifying the diagnostic value of modelling non-linear metabolite interactions. Box plot visualisation of the eight selected metabolites reveals why univariate analysis proved insufficient (Fig. 4). Individual features show modest or negligible between-group differences when examined in isolation, with many displaying poor univariate statistical significance. Permutation feature importance values shown in the figure demonstrate that diagnostic signal emerges through the collective pattern across multiple metabolites rather than from dominant individual markers—a synergistic metabolic signature only detectable through multivariate pattern recognition.

### Operating point performance

Table 2 shows the performance of the model at different operating points. Youden’s Index [48] (which balances sensitivity and specificity) and the clinically relevant screening- and diagnosis- optimised thresholds, showing that the model not only performs well in the balanced case but is especially adept for screening purposes (where sensitivity is 95% while specificity is still almost 70%).

## Discussion

Cystoscopy remains the gold standard for bladder cancer diagnoses, often enhanced by photodynamic diagnosis, however a study by Guldhammer et al. indicated that cystoscopy has a sensitivity of 81% and a specificity of 73%, suggesting a notable risk of missing or underdiagnosing bladder tumours [49]. These findings underscore the need for additional diagnostic methods alongside cystoscopy to improve accuracy and patient care [49]. Due to the invasive nature of cystoscopy various non-invasive technologies have been explored, including urine analyses based on the detection of mRNA, DNA, proteins and exfoliated cells [50]. Metabolomics, particularly focusing on VOCs, have shown promise in the detection of bladder cancer in recent years [16, 18, 32, 51]. Most of these studies applied the SPME method for VOCs extraction, with or without sample pretreatment. This exploratory study investigated the profile of urinary semi-volatile and VOCs using an optimised LLE method, which allows the analysis of compounds with higher molecular weights [26]. The results obtained for the classical statistics study (AUROC 0.752) were very similar to recently published work by Lett et al. [16] who achieved a sensitivity and specificity of 0.71 and 0.72 respectively, with an AUROC of 0.77, using an eight-VOC diagnostic biomarker panel, and SPME of acidified urine combined with GC-MS. In a recent study utilising HS-SPME coupled with GC-MS Carapito et al. [34] reported that an 8 VOC panel differentiated urine samples from bladder cancer patients from controls with an AUROC of 0.872, sensitivity 89% and specificity 92%. This study used 5 machine learning algorithms and found that Random Forest approaches gave the best diagnostic accuracy. The current study identified XGBoost as the best performing algorithm with an AUROC of 0.869. This highlights that despite different analytical methods the performance in separating bladder cancer from controls utilising machine learning with an 8 VOC panel is remarkably similar.

Table S2 summarises some of the VOC markers identified across GC based studies [15, 33, 34, 52] but includes one LC-MS based study [53]. There are 83 compounds listed as significantly altered in bladder cancer. The majority of the compounds are seen to be up-regulated in cancer in agreement with this study. Across the studies in Table S2 there were a number of compounds linked to oxidative stress including n-alkanes, branched alkanes, aldehydes and ketones. However, there is limited agreement regarding the exact VOC markers between individual studies. Nonanal was present in 4 studies [16, 33, 34, 52] although in the study of Lett et al. [16] it was decreased. Of the other shared compounds across studies 2-ethyl hexanol was present in 3 studies [16, 34, 52] and 2 methoxy phenol [16, 54] and 2-pentanone [33, 52], hexanal [32, 34], benzaldehyde [32, 53] and benzoic acid [32, 33] were present in two studies. There were no markers identified across the studies that were common with the current study. The study by Tyagi et al. did identify a number of long chain n-alkanes (undecance, dodecane, tetradecane, hexadecane) and a branched alkane to be associated with bladder cancer (Pentadecane,2,6,10,14-tetramethyl-), in agreement with this study (nonadecane and 2,6,10,14 tetramethylhexadecane). The LC-MS study undertaken by Niziol et al. identified palmitamide [53] a derivative of palmitic acid which was identified as significantly associated with bladder cancer in this study. A general observation is that the biomarkers identified in this study were predominantly higher molecular weight than those identified by headspace sampling (the predominant method used in the other GC-MS based studies). This likely reflects the solvent extraction method selected for this study which is not directly comparable with SPME or direct headspace sampling used across the majority of the previous GC-MS and GC-IMS studies. The LLE methodology was adopted to increase the total number of VOCs and extend the analysis to semi-volatile compounds to assess their ability to discriminate bladder cancer. Compounds such as palmitic acid may not be observed when using SPME sampling or a standard chromatographic column.

When looking at the machine learning approach, the most notable feature is the discordance between univariate statistical significance and multivariate diagnostic importance. Of the eight metabolites comprising the optimal diagnostic panel, six showed no univariate statistical significance (*p* > 0.05), and the single most important feature (VAR00047, permutation importance *p* = 0.141) was entirely non-significant when tested individually (*p* = 0.839). This feature displays virtually overlapping distributions between cancer and non-cancer samples when examined in isolation yet contributes more to diagnostic accuracy than any other metabolite when analysed within the multivariate framework.

This finding challenges the traditional biomarker discovery paradigm, which prioritises metabolites showing large individual differences between disease states. Our results demonstrate that bladder cancer’s metabolic signature resides not in dramatically altered individual compounds, but in subtle, coordinated changes across multiple metabolic pathways that manifest as complex, non-linear patterns. Features that appear to have limited diagnostic utility when examined individually become diagnostically significant when their relationships with other metabolites are considered. The preliminary analysis comparing healthy individuals who had never had bladder cancer versus patients in complete remission, and patients with newly diagnosed versus recurrent disease, revealed no detectable metabolic differences. This finding suggests that bladder cancer does not leave persistent metabolic signatures in urine after successful treatment, and that the metabolic profile of recurrent disease is indistinguishable from new diagnosis.

This observation has two important implications. First, the high diagnostic accuracy achieved when treating complete remission patients as non-cancer controls (rather than as a distinct intermediate category) validates their inclusion in the non-cancer group and suggests that successful treatment restores normal urinary VOC profiles. Second, the inability to distinguish new from recurrent disease based on metabolic profiles indicates that surveillance protocols need not differentiate between these populations when interpreting VOC-based tests—both represent active disease with similar metabolic manifestations.

GC-MS was combined with XC-MS, for feature detection, retention time alignment enabling rapid analysis of entire spectra - coupled with statistical methods for identification of significant ion fragments that differed between bladder cancer and controls. The retention times (RT), and m/z values for the VOCs used in the classical statistical model to separate bladder cancer samples from controls were:Var00002 = m/z70.05 and RT 33.68, Var 00004 = m/z 99.1 and RT 28.60, Var 00005 = m/z 113.1 and RT 28.60,Var 00007 = m/z 43.1 and RT 28.59, and RT 28.60,Var 00077 = m/z 50.1 and RT 35.54, Var 00108 = m/z 82.15 and RT 36.6 Var 00115 = m/z 70.05 and RT 30.94 and Var 00122 m/z 49.08, RT 36.6. All of these compounds were up-regulated in the bladder cancer samples. The 4 most significant features (Var 00002,00004,00005 and 00007) based on their retention times contain mass ions from only 2 distinct VOCs (RT 28.60 and RT 33.68). The best library match for these compounds was RT 33.68 - dodecyl acrylate, CAS no. 2156-97-0, (97% match) - this contains the ion fragment m/z 70 and RT 28.6 – nonadecane, CAS no. 629-92-5, (95% match) which contains the ion fragments m/z 43.1, 99.1 and 113.1. Library matches (>80%) for the significant m/z features at retention times 35.54, 30.94, 43.44 and 36.60 were not obtained. With higher molecular weight compounds the library matches are not exact and so it is likely that one of the compounds is a fatty ester and the other one a branched or straight chain alkane.

The retention times, and m/z values for the machine learning model were as follows Var00003 m/z 45.05, RT 30.58, Var 00010 m/z 127.05, RT 28.60, Var 00047 m/z 99.1, RT 30.60, var 00054 m/z 50.1, RT 33.72, Var00205 m/z 61, RT 52.28, Var 00258 m/z 94, RT 31.00, Var00274 m/z 73, RT 56.31 and Var 00813 m/z 67, RT 37.58. All of the compounds were up regulated in the cancer group vs. the control group with the exception of Var00274 and Var00813.

The tentative annotation of VOCs associated with the significant variables were as follows:

Var00003 and Var 00047 hexadecane 2,6,10,14-tetramethyl, CAS no. 504-44-9, (RT 30.58, m/z 85.05 and 99.1, 92.58% library match), Var00010 nonadecane (RT 28.60, m/z 127.05, 95.43% library match), Var00054 dodecylacrylate (RT 33.72, m/z 50.1, library match 96.69%), var00205 palmitic acid, CAS no. 57-10-3, (RT 52.28, m/z 61, library match 95.92%), Var00274 pyrrolo [1,2-a] pyrazine-1,4-dione hexahydro-3-(2-methylpropyl), CAS no. 5654-86-4, (RT 56.31, m/z 73, library match 88.6%). Library matches for the compounds Var00258 and Var00813 couldn’t be obtained.

Dodecylacrylate has been reported as a significant compound in urine samples from prostate cancer patients being upregulated in patients with a recurrence of prostate cancer [55]. Nonadecane has been identified previously in faeces and saliva of human subjects [15] and abnormal concentrations were associated with celiac disease [56]. 2,6,10,14-tetramethylhexadecane has not previously been found in the healthy human volatilome [15] or associated with disease states of humans.

Palmitic acid (hexadecanoic acid) has been identified in blood, faeces, sweat, saliva and urine in human subjects. Abnormal concentrations have been identified previously in breast [57] (serum), colorectal [58] (faeces) and bladder (urine) cancer [59]. Pyrrolo [1,2-a] pyrazine-1,4-dione hexahydro-3-(2-methylpropyl), CAS no. 5654-86-4, has not previously been identified in the healthy human volatilome [15] or in disease states of humans.

Palmitic acid is the most common saturated fatty acid in the human body accounting for 20–30% of total fatty acids [60]. The source can be exogenous from the diet or synthesised endogenously via *de novo* lipogenesis (DNL). Usually, palmitic acid has a steady state concentration in human tissues but in the presence of other factors such as excessive intake of carbohydrates (in particular mono and disaccharides), and a sedentary lifestyle, the mechanisms to maintain a steady state concentration may be disrupted causing over accumulation of tissue palmitic acid resulting in dyslipidemia, hyperglycemia, increased ectopic fat accumulation and increased inflammatory tone via toll-like receptor 4. Dodecylacrylate is most likely of exogenous origin as it is a monomer used in making polymers for adhesives, coatings and personal care products. Exogenous compounds are altered in cancer as the cancer cells and the body’s inflammatory response to the tumour means that compounds are metabolised differently than healthy tissues [61].

Nonadecane can have an exogenous source from the diet but can also be a product of the metabolic breakdown of fatty acids and phospholipids. Alkanes can be generated by various biological processes including oxidative stress and lipid degradation by the microbiota. Increased ROS levels associated with oxidative stress lead to DNA damage, protein oxidation, and lipid peroxidation, facilitating tumour initiation and advancement. Elevated markers of oxidative stress are consistently found in the serum and urine of bladder cancer patients [62]. There is also evidence that long chain alkanes act as co-carcinogens promoting cancer development in the presence of other carcinogens [63].

2,6,10,14-tetramethylhexadecane (phytane) is found in human adipose tissue and serum where it is present as part of the unsaponifiable fat fraction. The main source of phytane is the diet via the ingestion phytols, found in foods like ruminant fats (beef, lamb), dairy products, and certain fish. Different levels may be observed in cancer due to lipid metabolism reprogramming and alterations in the isoprenoid metabolism pathways (mevalonate pathway). Certain branched chain alkanes have also been seen to show high co-carcinogen activity [63].

Pyrrolo[1,2-a] pyrazine-1,4 dione, hexahydro-3-(2-methylpropyl) is a bioactive cyclic dipetide primarily of microbial origin. In humans it may be an extracellular metabolite of human probiotics such as *Saccharomyces cerevisiae*. The compound is known to have cytotoxicity against various cancer cell lines [64]. It is effective at inhibiting angiogenic factors and promoting apoptosis in cancer cells. Pyrrolo[1,2-a] pyrazine-1,4 dione, hexahydro-3-(2-methylpropyl) was one of the few compounds that was found in lower concentrations in the cancer group vs. the control group.

These compounds have only been tentatively annotated, and further work will be undertaken to identify them and validate them in future studies. The liquid-liquid extraction method was developed to increase the number of overall metabolites and increase the fraction of semi-volatile compounds. This meant that each chromatogram contained approximately 300 distinct VOCs (chromatographic peaks) and 918 distinct m/z values were identified by XCMS and used in the statistical modelling. A drawback of increasing the number of semi-volatile compounds is the poor library matches obtained for many of the larger molecular weight compounds. The poor library matches represent the number of possible molecules with overlapping fragmentation patterns but also highlights the limitations of mass spectral library databases. Although the NIST library is extensive we showed in previous work that over half the possible volatile organic compounds from the peroxidation of simple fatty acids were not included in the database [15, 65].

This also means that it would be difficult to run standards to confirm the identity of these compounds without targets from high quality library matches or matching retention indices etc. It is also likely for a range of higher molecular weight compounds that standards will not be commercially available.

### Classical statistics versus machine learning

Direct comparison between the classical statistical approach (volcano plot features with logistic regression; AUROC 0.752) and the machine learning approach (RFE-selected features with XGBoost; AUROC 0.869) reveals a 11.7 percentage point improvement in diagnostic accuracy (15.5% relative improvement). This performance differential cannot be attributed solely to feature selection strategy, as applying linear logistic regression to the RFE-selected features yielded only 0.652 AUROC—substantially worse than both the classical approach with its own features (0.752) and XGBoost with the same RFE features (0.869).

These results demonstrate two critical findings: 1. metabolites identified by univariate significance testing differ fundamentally from those identified by multivariate pattern recognition, and 2. diagnostic performance depends critically on the analytical framework’s capacity to model non-linear metabolite interactions. The volcano plot identifies individual markers with large effect sizes; RFE identifies metabolites whose collective pattern, rather than individual abundance, distinguishes disease states. When analysed through linear methods that assume independence or additive interactions, even optimally selected features underperform. Only when metabolite interactions are explicitly modelled through tree-based partitioning does the full diagnostic potential emerge.

This finding has implications for future biomarker discovery in metabolomics as the search for individual markers with dramatic univariate effects may not provide optimal performing models if the true biological signal resides in subtle, coordinated changes across multiple pathways that manifest as complex, non-linear patterns.

Despite the promising results, several limitations of this study should be acknowledged. The sample size was relatively small, which may limit the generalisability of the findings. The small sample size also limited the ability to stratify patients to assess whether the identified biomarkers (or different ones) were linked to disease severity. Larger, independent cohorts are needed to validate these results and ensure their robustness across diverse populations. In addition, any future study design should incorporate enough patients with early and late-stage cancers so a comparison between these two groups can be undertaken. The ideal case is for VOC markers to detect early-stage cancer or early recurrence in patients with previous diagnosis of bladder cancer.

While the LLE method allows for the analysis of higher molecular weight compounds, it may not capture the full spectrum of VOCs present in urine, potentially overlooking other relevant biomarkers particularly those that are lower molecular weight or with chemistry not aligned well with the extraction method. To enable clinical implementation of this method there would need to be work undertaken on effectively automating the liquid/liquid extraction of clinical urine samples. This has been undertaken for other types of standard clinical tests for example analysis of organic acids [66]. Once this was achieved a liquid autosampler on the front end of the GC-MS would enable high throughput of clinical samples. The resulting total ion chromatograms could be pre-processed and run automatically against the validated machine learning model.

The study’s cross-sectional design limits the ability to infer causality or temporal relationships between the identified biomarkers and bladder cancer progression. Future studies could adopt a longitudinal design to address these shortcomings and assess the biomarkers effectiveness for mapping cancer progression.

Additionally, while machine learning techniques improved diagnostic accuracy, the complexity and computational requirements of these models may pose challenges for clinical interpretability. This can be partially attenuated using permutation feature importances. Finally, the potential for overfitting in machine learning models must be carefully managed, particularly when dealing with high-dimensional data and relatively small sample sizes.

The model’s performance at different operating points demonstrates versatility for various clinical contexts. At the screening-optimised threshold, sensitivity of 95.2% would detect nearly all cancer cases whilst maintaining specificity of 69.6%—comparable to or exceeding conventional urine-based bladder cancer tests such as cytology (sensitivity 40–60%) or FDA-approved protein markers (sensitivity 50–90%, specificity 60–90%). The non-invasive nature of VOC analysis from untreated urine, combined with potential for point-of-care deployment via portable mass spectrometry or electronic nose technologies, positions this approach favourably for screening applications, particularly in high-risk populations (e.g., occupational exposures, prior cancer history) or haematuria workup.

At the diagnosis-optimised threshold, specificity of 93.2% minimises false positives, reducing unnecessary cystoscopies—an invasive, expensive procedure with patient burden and complication risks. A VOC-based test could serve as an initial triage, reserving cystoscopy for test-positive individuals. With sensitivity of 66.2% at this threshold, approximately one-third of cancers would be missed; however, in symptomatic patients or surveillance protocols where repeat testing occurs, cumulative sensitivity across multiple time points may prove acceptable, particularly if the test detects aggressive disease preferentially (a hypothesis requiring validation).

The confidence-based prediction analysis suggests a tiered deployment strategy. High-confidence predictions (90.7% accuracy, 49.6% of cases) could drive immediate clinical decisions, whilst low-confidence predictions (70.0% accuracy, 18.0% of cases) trigger reflex confirmatory testing or alternative diagnostic pathways. This risk-stratified approach mirrors current clinical practice for other biomarkers and may improve cost-effectiveness and patient outcomes compared to uniform threshold application.

## Conclusions

This study showed that urinary volatile organic compounds provided a powerful metabolic signature for bladder cancer detection, achieving AUROC exceeding 0.85 (95% sensitivity, 70% specificity, optimised for screening) when analysed through machine learning frameworks that model non-linear metabolite interactions. Critically, diagnostic signals reside in subtle, coordinated patterns across multiple VOCs rather than in dramatically altered individual compounds, a signature invisible to conventional univariate statistical approaches. These findings challenge the traditional biomarker discovery paradigms that prioritise individual markers with large effect sizes and demonstrate that high-dimensional metabolomic data require analytical frameworks specifically designed to capture complex feature interactions. With further validation in independent cohorts and development of simplified analytical platforms, VOC-based testing may provide a non-invasive, rapid, and cost-effective complement or alternative to cystoscopy for bladder cancer detection, particularly in screening, haematuria workup, and surveillance applications.

## Supplementary information

Co-morbities tableS1 and compounds TableS2finalbc (1)

## Data Availability

The data relevant to the current study are available from the corresponding author on request.
