# Supplementary material for: Urinary volatilomics using liquid-liquid extraction and gas chromatography-mass spectrometry (GC-MS) combined with machine learning algorithms as a tool for diagnosis and surveillance of urothelial bladder cancer
Source: BJC Rep. 2026 Aug 7;4:42. doi: 10.1038/s44276-026-00244-8 (PMC13451427; doi:10.1038/s44276-026-00244-8)
Supplement: Supplementary file 1 [file 44276_2026_244_MOESM1_ESM.docx]

**Table S1.** Listed co-morbidities of participants in this study

|  | **Cancer** |  | **Control** |  |
| --- | --- | --- | --- | --- |
|  | **New UBC diagnosis** | **Recurrence of UBC** | **non-UBC haematuria** | **no recurrence of UBC** |
| Number of participants |  |  |  |  |
| General | 15 | 35 | 30 | 20 |
| No co-morbidities listed | 10 | 31 | 11 | 15 |
| Co-morbidities listed for remaining patients | Hypercholesterolaemia , IBS , Supraspinatus , neutropenia | Brain tumour , Dementia | Fatty liver, carpal tunnel syndrome, Dupuytrems contracture, Diverticular disease, dermofasciectomy, hiatus hernia, depression | Stroke |
|  | Vitamin D deficiency, psoriatic arthritis , rheumatoid arthritis , gout , pneumonia | Wheat allergy , drug induced urticaria, detrusor instability , GORD | Spinal stenosis, vitamin D deficiency, osteoperosis, anxiety, depression | IHD, aortic stent, quad bypas graft |
|  | Asthma, Basal cell carcinoma, stress incontinence, essential hypertension, arthritis | Diabetes, Atrial septal defect, pacemaker | Fracture T12 vertebra, osteopenia, osteoperosis, lumbar spine stenosis, mistral regurgitation, coronary artery disease, essential hypertension, chronic gastritis, cardiac catheterisation, hysterectomy | Hypertension, Back pain |
|  | Diabetes, Hypertension, Transient ischaemic attack, Abnormal liver function | T2 N0M0 lung cancer surgically resected | Protein deficiency, urethral structure, prostatism, bladder flow obstruction, urethral dilation, sciatica, shingles, right insuinal hernia, polyp, blanopostitis | Diabetes , TCC left renal pelvis 2006 |
|  | CKD3, Bladder diverticulum, osteopenia, pneumonia |  | Hernia, depression IBS | Diabetic |
|  | Metastatic breast cancer 2015 |  | Hep C pos, COPD, Drug user | Metastatic breast cancer 2015 |
|  |  |  | Hypertension, Hypothyroidism |  |
|  |  |  | UTI |  |
|  |  |  | Hypertension, Left ventricular hypertrophy, anaemia, CKD, recreational amphetamine drug user |  |
|  |  |  | ISO Nephropathy, CKD Stage 36 A1, Previous AKI + cardiomyopathy, hyperlipidaemia, pleurisy, angina, angiogram |  |
|  |  |  | Known prostate cancer- Radiotherapy 2013 |  |
|  |  |  | Previous colon cancer |  |
|  |  |  | Testicular lump, Dysuria, Biz deficiency, CKD 3, COPD, Rheumatoid arthritis, Coronary heart bypass, Angina. |  |
|  |  |  | Hypertension, osteopenia, high cholesterol |  |
|  |  |  | Previous AF, DC cardioversion, previous ablation, Gout, HTN, Sleep apnoea |  |
|  |  |  | Chronic Pancreatitis, Cholelithiasis, Gall stones |  |
|  |  |  | Hypertension, IHD, High cholesterol |  |
|  |  |  | Corns, high cholesterol, PE hypertension, rectal resection |  |
|  |  |  | Hypothyroidism, AF, Hip Replacement, COPD, OA, Hypertension |  |
|  |  |  | Aortic Aneurysa, basel cell carcinoma, microlytic hypochronomic aneamia, waldenstroms macroglobulinaemia, lymphoma, coeliac disease |  |
|  |  |  | Hysterectomy, fractured pelvis, urethal dialation, flexi cystoscopy, osteoperosis, UTIs, glaucoma, Icchaemic heart disease, bronchiectossis |  |
|  |  |  | AF , pernicious anaemia , peripheral neuropathy , peritonitis , appendectomy , gastrectomy , Basal cell carcinoma , OA , Colostomy , Gallstones , tinnitus , bursitis , hernia repair , adenocarcinoma |  |

**Table S2**. Compounds linked to Bladder cancer in previous studies

| Study | Compounds | Analytical method |
| --- | --- | --- |
| This study | - Hexadecane 2,6,10,14-tetramethyl (increased) - Nonadecane (increased) - dodecylacrylate (increased) - palmitic acid (increased) - pyrrolo [1,2-9] pyrazine-1,4-dione hexahydro-3-(2-methylpropyl) (decreased) | Solvent extraction coupled with GC-MS |
| Ligor, T.; Ada  mczyk, P.; Kowalkowski, T.; Ratiu, I.A.; Wenda-Piesik, A.; Buszewski, B. Analysis of VOCs in Urine Samples Directed towards of Bladder Cancer Detection. *Molecules* **2022**, *27*, 5023. https://doi.org/10.3390/molecules27155023 | - butyrolactone (increased) - 2-methoxyphenol (increased) - 3-methoxy-5-methylphenol (increased) - 1-(2,6,6-trimethylcyclohexa-1,3-dien-1-yl)-2-buten-1-one (increased) - nootkatone (increased) - 1-(2,6,6-trimethyl-1-cyclohexenyl)-2-buten-1-one.(increased) | Head space solid phase microextraction (SPME) gas chromatography time of flight mass spectrometry (GC×GC TOF MS) |
| Â. Carapito, V.S. Fernandes Ferreira, A.C. Silva Ferreira, A. Teixeira-Marques, R. Henrique, C. Jerónimo, A.C.A. Roque, F. Carvalho, J. Pinto, P. Guedes de Pinho,  Non-invasive bladder cancer detection: Identification of a urinary volatile biomarker panel using GC-MS metabolomics and machine learning,  Talanta,  Volume 297, Part B,  2026,  128749,  ISSN 0039-9140,  https://doi.org/10.1016/j.talanta.2025.128749. | - 4-methyl-2-heptanone (increased) - 4-methyl-2-pentanone (increased) - acetone (downregulated) - nonanal (increased) - hexanal (increased) - 2,5-dimethylbenzaldehyde (increased) - 2-ethyl-1-hexanol (increased) - p-cresol (increased) | headspace solid-phase microextraction coupled with gas chromatography–mass spectrometry (HS-SPME/GC-MS) |
| Mao, M., Zhang, Y., Liu, H. *et al.* Identification of a panel of volatile organic compounds in urine for early detection of for bladder cancer. *Sci Rep* **15**, 17383 (2025). https://doi.org/10.1038/s41598-025-01324-4 | - 2-Undecenal (increased) - 2-propanone (increased) - 1-octanal (increased) - Ethyl 2-hydroxybenzoate (increased) - 2-ethyl hexanol-D (decreased) - 2-Pentanone (increased) - 1-Propanol-3-(methylthio) (increased) - 1-nonanal (increased) | gas chromatography-ion mobility spectrometry (GC-IMS) |
| Lett, L., George, M., Slater, R. *et al.* Investigation of urinary volatile organic compounds as novel diagnostic and surveillance biomarkers of bladder cancer. *Br J Cancer* **127**, 329–336 (2022). <https://doi.org/10.1038/s41416-022-01785-8> | - Nonanal (Decreased) - 2-ethylhexan-1-ol (Decreased) - 1,1,4a-trimethyl-4,5,6,7-tetrahydro-3Hnaphthalen-2-one (Decreased) - 5-ethyl-3-methyloxolan-2-one (Increased) - Phenol (Increased) - 4-methylpent-3-enoic acid (Increased) - 2-methoxyphenol (Increased) - 3-methylheptan-2-one (Increased) - 1,2,4,5-tetramethylbenzene (Increased) - Heptan-2-one (Increased) | solid-phase microextraction and VOC analysis applied gas chromatography-mass spectrometry |
| Nizioł, J., Ossoliński, K., Płaza-Altamer, A. *et al.* Untargeted urinary metabolomics for bladder cancer biomarker screening with ultrahigh-resolution mass spectrometry. *Sci Rep* **13**, 9802 (2023). https://doi.org/10.1038/s41598-023-36874-y | - 2,5-Furandicarboxylic acid - 2-Furoylglycine - 3,4-Dihydroxymandelic acid - Azelaic acid - Benzaldehyde - Choline - Cis,cis-Muconic acid - Indoleacetic acid - Isostearic acid - Methylmalonic acid - Mevalonic acid - N-Acetylserotonin - N-Alpha-acetyllysine - Oleamide - Palmitamide - Pantothenic acid - Phenylacetylglycine - Phenylglyoxylic acid - Picolinuric acid - Sebacic acid - Succinic acid - Vanillic acid | ultra‑high‑performance liquid chromatography and ultra‑high‑resolution mass spectrometry |
| Tyagi, H.; Daulton, E.; Bannaga, A.S.; Arasaradnam, R.P.; Covington, J.A. Urinary Volatiles and Chemical Characterisation for the Non-Invasive Detection of Prostate and Bladder Cancers. *Biosensors* **2021**, *11*, 437. https://doi.org/10.3390/bios11110437 | - Biphenyl - Nonanal - Tetradecane - Pentadecane,2,6,10,14-tetramethyl- - 2-Pentanone - Undecane - 4-Heptanone - Dodecane - Hexadecane - Heptanal - MethylIsobutylKetone - Naphthalene - Benzoic acid | gas chromatography-ion mobility spectrometry (GC-IMS) and GC-TOF-MS |
| Cauchi M, Weber CM, Bolt BJ, Spratt PB, Bessant C, Turner DC, et al. Evaluation of gas chromatography mass spectrometry and pattern recognition for the identification of bladder cancer from urine headspace. *Analytical Methods*. 2016;8(20): 4037–4046. https://doi.org/10.1039/C6AY00400H. | - 2-pentanone (decrease) - 2,3-Butanedione (decrease) - 4-heptanone (decrease) - Dimethyldisulphide (decrease) - Hexanal (increase) - Benzaldehyde (increase) - Butyrophenone (increase) - 3-Hydroxyanthrnilic acid (increase) - Benzoic acid (increase) - Trans-3-hexanoic acid (increase) - Cis-3-Hexanoic acid (increase) - 2-butanone (increase) - 2-propanol (decrease) - Acetic Acid (decrease) - Piperitone (decrease) - Thujone (decrease) | SPME -Headspace GC-TOF-MS |
